# Do applicants from Generation X, Y, Z differ in personality traits? data from selection procedures in aviation (1987–2019)

**DOI:** 10.3389/fpsyg.2023.1173622

**Published:** 2023-08-01

**Authors:** Dirk Stelling

**Affiliations:** Department of Aviation and Space Psychology, Institute of Aerospace Medicine, German Aerospace Center (DLR), Cologne, Germany

**Keywords:** generations, personality, selection, aviation, self-presentation, gender, age

## Abstract

**Introduction:**

The objective of this study is to research personality trait differences across generations and the impact of age, gender and self-presentation on these traits.

**Methods:**

A total of 82,147 applicants (aged 17–24) for aviation training (pilot, air traffic controller), born between 1965 and 2002, were divided into three cohorts (Generation X, Y, Z). We analysed data from the *temperament structure scales* (TSS) personality questionnaire, which was collected during selection procedures between 1987 and 2019. Generational differences were analysed by ANCOVAs with generation and gender as group factors, controlled by age and self-presentation (social desirability).

**Results:**

Age had no significant impact, but we observed slight gender differences in emotional stability, vitality, empathy, and self-presentation across all generations. The generational differences found exhibited extremely small effect sizes, suggesting that applicants have become more extraverted, controlled (with lower aggression and higher rigidity), and inclined to present themselves in a more favourable manner.

**Discussion:**

We discuss the implications of these findings for the aviation industry and the applicability of Generation theory in personality trait research.

## 1. Introduction

Certain personality traits such as extraversion, neuroticism, self-confidence, conscientiousness, and agreeableness have demonstrated significance within the aviation field (Barron et al., 2016; Breuer et al., 2023). Generational theory predicts changes in these personality traits for individuals who were born in a specific period of time (refer to 1.1). Costanza et al. (2017) observe that there is “…*a growing sense among a group of authors, consultants, trainers, and management gurus that there are substantive and meaningful generational differences between individuals in today’s workplaces*.” Djabi and Shimada (2017), in their meta-analysis, discovered a growing interest in generational diversity and its impact on work, resulting in an increase in publications.

Given the impending mandatory retirement of a significant percentage of pilots, the aviation industry is now interested in the generational shift in the workforce and its implications for training and safety (Birdsong and Reesman, 2023). However, do people from different generations really differ in personality traits?

### 1.1. Generational theory

If the environment in the form of historical or social events, influences individuals within a particular age range, they are classified as belonging to a generation (Mannheim, 1928). Authors may differ in the names assigned to generations and their specific birth time periods, but most of them distinguish baby boomers—born 1944 to 1960-, Generation X—born 1961 to 1980 and Generation Y—born 1981 to 2000—(Arsenault, 2004). In recent publications a digital grown up Generation Z—born from 1995 to now—distinct from Generation Y (Seemiller and Grace, 2017). Shared experiences and the development of a collective memory should end up in a shared habitus, common attitudes, values and beliefs (Arsenault, 2004).

Generational theory not only describes differences between generations, but summarizes environmental factors and predicts their impact on values, attitudes, norms and personality traits. For instance, Whitney Gibson et al. (2009) outlined several reasons for differences among baby boomers (economic prosperity after WWII, social changes in the 1960s), Generation X (both parents working, high divorce rates, corporate downsizing, AIDS epidemic, end of the Cold War), and Generation Y (upbringing with cell phones and MTV, 9/11, computer games, and social networks). Generation Z finally is raised immersed in the internet, is at home in the real and digital world at the same time, expects diversity and presents themselves in the social media (Lanier, 2017). Other important reasons for the difference of generations include increased mobility, a less rule bounded parenting style and a general shift towards a service-oriented society (Twenge, 2001a). Simultaneously, the modern world is associated with reduced social connectedness and an increased perception of environmental threats (Twenge, 2000).

### 1.2. Differences in personality traits predicted by generational theory

Numerous studies on generational differences focus on workplace behaviour and attitudes. Generation Yers are reported to exhibit higher levels of self-enhancement compared to Generation X (Lyons et al., 2007), and discussions have arisen regarding different motivations for working overtime between generations (Becton et al., 2014). However, some authors also report variations in personality traits across generations (see Table 1). Results for openness to experience are inconclusive (Smits et al., 2011), but extraversion is generally associated with an increase over generations due to enhanced mobility, less rigid parenting styles, and a shift towards a service-oriented outlook (Twenge, 2001a). Effects are reported for specific subpopulations (André et al., 2010) or entire generations, with either small (Smits et al., 2011) or large (Twenge, 2001a) effect sizes.

**Table 1:** Empirical results of cohort differences for several personality traits, their link to generation and the period of testing.

Personality traits related to …	Trend	Generation	Period of testing	Cohort	Publication
*Emotions*					
Anxiety, neuroticism	Increase	BB, X	1952–1993	Students, children	Twenge (2000)
Neuroticism	Increase	Y, Z	Pre 2027	Students	Caganova et al. (2017)
Depression	Increase	BB-Y	1960s–2007	Students	Twenge and Campbell (2008)
*Extraversion*					
Extraversion	Increase	X, Y, Z	1991–2010	Students	Twenge (2001a)
Extraversion	Increase	X, Y	1982–2007	Students	Smits et al. (2011)
*Inter-personality*					
Narcissism, self-esteem	Increase	BB-Y	1960s–2007	Students	Twenge and Campbell (2008)
Agreeableness	Increase	X, Y	1982–2007	Students	Smits et al. (2011)
Empathy	Decrease	BB, X, Y	1979–2009	Students	Konrath et al. (2011)
*Performance*					
Conscientiousness	Increase	X, Y	1982–2007	Students	Smits et al. (2011)
Perfectionism	Increase	X, Y	1989–2016	Students	Curran and Hill (2019)
Achievement assets, impulse control	Increase	BB, Y	Pre 1987, 2004–08	Students	Stewart and Bernhardt (2010)
*Gender*					
Masculinity	Increase	BB-X	1975–1994	Women	Twenge (1997)
Assertiveness, dominance	Increase	Before BB-X	1931–1993	Women	Twenge (2001b)
Aggression	Increase	Silent-X	1968/69 2004/05	Women	André et al. (2010)

Neuroticism is reported to increase over generations as a consequence of less social connectedness and a rise in environmental dangers (Twenge, 2000). For example, Twenge (2000) observed a rise in anxiety and neuroticism among Americans. Upper scores of neuroticism are also reported for Generation Z compared to Generation Y (Caganova et al., 2017). Subsequent generations seem to have higher levels of depression (Twenge and Campbell, 2008), aggressive non-conformance (André et al., 2010) and lower scores in impulse control (Stewart and Bernhardt, 2010). Different results are reported by Smits et al. (2011), who described only a slight linear decrease in neuroticism in a Netherland’s student cohort. Also a higher level in self-confidence is reported in today’s students (Twenge et al., 2012b).

Some studies report an increase in self-orientation across generations, with individuals becoming more narcissistic (Twenge et al., 2008; Stewart and Bernhardt, 2010), more individualistic (Blok, 1998; Twenge, 2010) and less concerning about others (Twenge et al., 2012a). The use of personal technology and media in everyday life is assumed to be a contributor to declining empathy, especially in samples after 2000 (Konrath et al., 2011). On the other hand, studies report a small linear increase in agreeableness (Smits et al., 2011) or showed no (Trzesniewski et al., 2008) or very small effect sizes for differences in narcissism across generations (Donnellan et al., 2009).

### 1.3. Additional reasons for cohort differences

Differences in certain personality traits between cohorts may be explained by variations in the proportions of men and women within those cohorts. Some findings in generational research suggest, that certain differences hold true only for female cohorts (see Table 1). It is well-known that there are differences in interests between men and women (Lippa, 2010), but when it comes to personality traits, the distributions largely overlap (Weisberg et al., 2011). However, there are small yet consistent differences. On average, women tend to have higher levels of neuroticism, conscientiousness, agreeableness, extraversion (Lippa, 2010; Vecchione et al., 2012; Lehmann et al., 2013), and various facets of empathy (Davis, 1980). On the other hand, men tend to have higher levels of openness to experience (Vecchione et al., 2012; Lehmann et al., 2013) and a greater inclination towards seeking thrill and adventure (Rahmani and Lavasani, 2012). Normally the effect sizes are moderate to small, depending on type of measurement used (Vianello et al., 2013). Given these results and the possibility of differing gender proportions within cohorts, we chose to account for gender differences as a primary factor in our study.

Across the lifespan, changes in values (Kalleberg and Marsden, 2019) and traits can be observed (Lucas and Donnellan, 2011; Specht et al., 2011). Traits tend to be relatively stable over shorter periods during adulthood (Terracciano et al., 2010; Cobb-Clark and Schurer, 2012). However, intra-individual developments have been reported from adolescence to emerging adulthood, with variations observed between genders (Vecchione et al., 2012). Wong et al. (2008) suggest that differences in traits may be more closely related to age than to generation. Therefore, even when studying only young adults, age is a variable that should be controlled in research studies.

### 1.4. Problems in measuring generational differences

Untangling the effects of age, measurement period, career stage, and cohort in contributing to generational differences is challenging (Parry and Urwin, 2011). Many findings are based on cross-sectional designs, where data is collected at a single point in time from individuals of different ages representing different generations. These studies overlook the possibility that age might be the actual cause of trait differences (Costanza et al., 2017). It is also possible that variations in values, such as work values, are more influenced by the period of measurement rather than the date of birth (Kalleberg and Marsden, 2019). A limitation of some studies is that results are based on aggregated data from relatively small samples, using sample means instead of individual data points (Trzesniewski et al., 2008). Furthermore, it is criticized that in most studies, observed cohort differences are interpreted as changes in latent variables without controlling for measurement invariance (Smits et al., 2011). Even evaluating one database using different statistical methods can lead to different conclusions about generations (Costanza et al., 2017). Many empirical results are inconsistent or not sufficiently robust, and there may be more variation in personality traits among individuals within generations than between generations (Dencker et al., 2008; Donnellan and Trzesniewski, 2009).

Another challenge in exploring generational differences lies in the methodology of questionnaires themselves. It is known that questionnaire results can be influenced by response styles (van Herk et al., 2004), and socially desirable responding can compromise the validity of self-report measures, particularly in high-stakes situations (Bou Malham and Saucier, 2016). Impression management, the tendency to create or maintain a certain self-image, is a current topic of research (Bolino et al., 2016), and response bias can contribute to variations in trait dimensions (Morales-Vives et al., 2017). In the field of aviation and space, we have observed an impact of self-presentation on variables related to emotional stability and conscientiousness during selection procedures (Goeters et al., 1993; Mittelstädt et al., 2016). What if there are changes in self-presentation across generations? Twenge et al. (2012b) report data from 1966 to 2009, indicating that more students rated themselves as above average in various abilities compared to previous generations. Thus, differences in self-description between generations could reflect true shifts in traits, changes in social desirability regarding specific traits (e.g., extraversion), or the intention to portray oneself in line with a particular image (Twenge, 2001a).

### 1.5. Measuring personality traits in German applicants for aviation jobs

In this study, we compared differences between Generation X, Y, and Z in personality traits measured by a personality questionnaire (TSS) that we used during our selection procedures. The TSS, developed by DLR, has its roots in the 1970s prior to the widespread popularity of the Big Five framework (Costa and McCrae, 1992). The description of all scales, is presented in Table 2.

**Table 2:** Description of low and high scores in the temperament structure scales (TSS).

TSS scales		Low score	High score
Instability (emotional)	INS	Resilient, optimistic	Nervousness, easily frustrated
Aggressiveness	AGG	Peaceable, diplomatic	Impulsive, obstinate
Extraversion	EXT	Reserved, does not mind being alone	Sociable, lively
Mobility	MOB	Low local mobility, avoids any risks	Ready to take risks, seeks changes
Vitality	VIT	Soft, low interest in physical fitness	Robust, active in sports
Achievement	ACH	Avoids effort, enjoys life	Ambitious, always busy
Rigidity	RIG	Spontaneous, no sense of order	Tactical, principle minded
Empathy	EMP	Rational, hard-hearted	Sympathetic, altruistic
Dominance	DOM	Unpersuasive, avoids leadership	Decisive, likes to be the leader
Spoiltness	SPO	Unpretentious, needs no luxury	Highly demanding, lavish
Openness	OPN	Denies own weakness, always ideal behaviour	Admits weakness, admits non-conformist behaviour

Some scales can be recognized as factors (Extraversion, Emotional Instability) or facets (Achievement, impulsive Aggressiveness, Dominance) within the Big Five framework, while other scales were developed specifically for the selection of trainees in aviation jobs. Traits such as being structured yet flexible (Rigidity), adaptability to change (Mobility), maintaining physical fitness and resilience (Vitality), exhibiting compassionate teamwork (Empathy), or coping with constraints (Spoiltness) reflect the demands necessary during training and on the job. The TSS scale for Openness measures social desirability and self-presentation and should not be confused with the Big Five factor of Openness to experience. Mittelstädt et al. (2016) provide an integration of all these scales within the Big Five concept.

The original form of the TSS used in our analysis has remained unchanged over the years and has demonstrated varying but acceptable levels of internal consistency for all scales (Table 3). Various versions of the TSS have shown predictive validity in pilot selection (Stahlberg and Hoermann, 1993; Hörmann and Maschke, 1996; Guan et al., 2003), as well as its application in air traffic control (Pecena et al., 2013) and astronaut selection (Maschke et al., 2011).

**Table 3:** Published scores of internal consistency (Cronbach’s alpha) for the German version of the temperament structure scales (TSS).

TSS scales		1987^a^ *N* = 288/284	1993^b^ *N* = 300	2016^c^ *N* = 249
Instability (emotional)	INS	0.83–0.85	0.79	0.79
Aggressiveness	AGG	0.75–0.76	0.61	0.80
Extraversion	EXT	0.84–0.87	0.67	0.80
Mobility	MOB	0.85–0.87	0.84	0.81
Vitality	VIT	0.81–0.84	0.85	0.87
Achievement	ACH	0.71–0.77	0.61	0.72
Rigidity	RIG	0.84–0.87	0.74	0.80
Empathy	EMP	0.85–0.86	0.63	0.76
Dominance	DOM	0.85–0.85	0.82	0.81
Spoiltness	SPO	0.79–0.83	0.87	0.69
Openness	OPN		0.83	0.76

a Maschke (1987).

b Goeters et al. (1993).

c Mittelstädt et al. (2016).

Maschke (1987) reports numerous statistical analyses on the TSS. In two different samples, he found average retest reliabilities of the scales to be 0.82 after two to four months and 0.52 after 6 years. There were significant correlations (0.16–0.63) between the TSS scales and self-assessments as well as biographical and other data from application documents (0.19–0.56). The TSS exhibits construct validity when compared with other personality questionnaires (see summary in Mittelstädt et al., 2016) and demonstrates moderate correlations with questionnaires assessing social competence (Hörmann et al., 2007).

### 1.6. Hypothesis

Based on the generational theory and empirical findings mentioned above, we expect:

*H1*: Emotional *Instability* increases from Generation X to Z.

*H2*: *Aggressiveness* (related to Agreeableness and impulse control) decreases from Generation X to Z.

*H3*: *Extraversion* shows an increasing trend from Generation X to Z.

*H4*: *Mobility* increases from Generation X to Z.

*H5*: Physical fitness became more important and *Vitality* increases from Generation X toY, Z.

*H6*: *Achievement* increases from Generation X to Y, Z.

*H7*: *Rigidity* (as a facet of conscientiousness) increases from Generation X to Z.

*H8*: Generation Z is the most egocentric and shows less *Empathy* then X, Y.

*H9*: *Dominance* increases from Generation X to Z.

*H10*: Higher levels of narcissism lead to increased levels of *Spoiltness* from Generation X to Z.

*H11*: Self-presentation is highest in Generation Z with the highest scores in the *TSS Openness* scale.

*H12*: Women show higher levels of (emotional) *Instability*, *Extraversion*, *Empathy* and lower levels of *Mobility*. In terms of generations, they differ from men in the development of *Dominance* and *Aggressiveness*.

## 2. Materials and methods

### 2.1. Participants

The sample comprised of 82,147 men and women who were applying for aviation training (pilot, air traffic controller). The age range was restricted from 17 to 24 years at the time of testing, with applicants born between 1965 and 2002. The testing was conducted at the facilities of the German Aerospace Centre (DLR) in Hamburg, Germany, between 1987 and 2019.

All applicants held German citizenship, and only test campaigns conducted in the German language were included in the evaluation. Our data was collected during the initial stage of the selection process, which involved group testing of abilities, knowledge, personality, and English language proficiency. The temperament structure scales (TSS) were integrated into the selection procedure. The sample was limited to applicants who were participating in a selection procedure for the first time and held leaving certificates ranging from German secondary school (after 10th grade) to German university entrance level (after 12th or 13th grade). We followed the generational concept (refer to Section 1. Introduction) and categorized the sample into cohorts known as Generation X (born 1965–1980), Y (born 1981–1994), and Z (born 1995–2002).

### 2.2. Method

The TSS had various versions designed to fit the age, language, and cultural background of applicants. For this study, we utilized the German version developed for school leavers without specific aviation experience. The version used consisted of 180 items (15 per scale) and included a social desirability control scale called Openness, which comprised 30 items. The respondents used a forced two-choice (yes/no) answering format for statements provided or selected one of two alternatives for self-descriptions. The items have remained unchanged since 1987, enabling the use of comparable raw scores for each scale.

In 2000, the pencil-paper booklet version was transitioned to a computer-based format. The answer schema and items remained identical, and the test administration process was comparable to the booklet version. We examined data from 1 year prior to and after the transition and found no differences between computer-based and pencil-paper presentations.

The TSS were naturally not primarily designed for comparing generational differences. However, they are also suitable for this purpose. It encompasses the two temperament factors of the Big Five (Extraversion, Emotional stability), facets of Conscientiousness (Achievement, Rigidity), and additional areas such as Empathy, Mobility, Dominance, and Self-presentation, which are reported on in terms of generational differences.

### 2.3. Procedure

In each generation, the TSS was presented as part of the selection test battery, with its outcomes influencing the diagnostic decisions. All items were required to be answered, and there was no strict time limit, unlike the ability or knowledge tests. Typically, the test was administered before a lengthy lunch break to allow participants the opportunity to complete the test during the break time.

The pencil-paper version was scanned using an optical document reader, while the computerized version was processed using statistical programs. Raw scores for each variable were compiled along with biographical data, testing dates, and other test results in a SQL database in accordance with data protection regulations. Only data where no abnormalities were documented in the test protocol were included in our analysis.

We did not have information about the socioeconomic status of our applicants. However, in each generation, the educational background was consistent, as all participants held German citizenship and were German-speaking school leavers seeking training as pilots or air traffic controllers. In our study, we used biographical variables such as date of birth, sex, and citizenship to establish the study cohorts, and age at testing, year of testing, and the Openness scale were used as control variables.

### 2.4. Analysis

All statistical analyses were conducted using SPSS 21 software.^1^ We performed a stanine transformation on the raw data of the entire sample to standardize all TSS variables on a common scale and improve the fit to a normal distribution. The transformed data for each scale were used for analysis.

Due to the unavailability of item-level data for pencil-paper and pre-2000 computer data, we were unable to assess structural invariance.

The focus of this study was on analyzing differences between three generations rather than general trends. Therefore, we opted against conducting an age-period-cohort analysis and instead chose to directly compare the three groups. To analyze generational differences (H1–H12), we conducted a two-way ANCOVA with generation and gender as group factors, and age and self-presentation (Openness from TSS) as covariates. Since there was confusion between generation and year of testing, we examined the influence of the year of testing variable as a covariate and decided to exclude it from the analysis.

We examined main effects, interactions, and the impact of each control variable. We also conducted analyses using raw data and found no differences in results compared to stanine scores. Due to the large sample size, all effects were highly significant. Therefore, we used omega square (*ω*^2^) as a conservative measure of effect size (Okada, 2013). According to Cohen (2013), we defined *ω*^2^ as a small effect (0.01–0.059), medium effect (0.06–0.139), or large effect (>0.14), and Pearson correlation between the TSS-*Openness* scale and other scales as small (0.10–0.29), medium (0.30–0.49), or large (>0.50).

## 3. Results

### 3.1. Descriptive statistics

Table 4 presents the descriptive statistics for the cohorts. Generation Y is the largest group, but there are sufficient applicants in all other cohorts for statistical analysis. The percentage of women increased from 19% in Generation X to 28% in Generation Y and Z. This reflects the changing nature of the aviation industry, which has become increasingly attractive to women. While the educational background is comparable across all samples, the mean age of applicants decreased from 20.94 years in Generation X to 18.84 years in Generation Z. This decrease is particularly pronounced among men and can be attributed to changes in regulations. Germany abolished mandatory military and civil service for men, allowing them to apply directly after completing school. Additionally, changes in the school system enabled some students to reach the university entrance level 1 year earlier. Although we focused on young adults in each generation, we used age at the time of testing as a control variable.

**Table 4:** Descriptive statistics for the cohorts (total *N* = 82,147).

	Generation X		Generation Y		Generation Z	
	Male	Female	Male	Female	Male	Female
*n*	16,289	3,814	36,547	13,984	8,338	3,175
%^a^	81	19	72	28	72	28
*Age*						
*M*	21.16	19.98	19.98	19.49	18.82	18.83
SD	1.41	1.59	1.49	1.48	1.42	1.45
*M*	20.94		19.84		18.84	
SD	1.52		1.51		1.43	
UEL^b^	96.7%		99.6%		99.1%	
Birth range	1965–1980		1981–1994		1995–2002	
Test period	1987–1998		2000–2019		2012–2019	

a Percentage in cohort.

b UEL, university entrance level (German Abitur).

### 3.2. Generational differences

Table 5 displays mean and standard deviation of all TSS variables, split for gender. The covariate age had no effect [all *F*(1,821,389) < 543. 68, *ω*^2^ < 0.01] so we will present the results limited to *Generation*, *Gender* and *Self-presentation*.

**Table 5:** Descriptive statistics for TSS scales for Generation X, Y, and Z, split for gender.

	Generation X				Generation Y				Generation Z			
	Male		Female		Male		Female		Male		Female	
	*n =* 16,289		*n =* 3,814		*n =* 36,547		*n =* 13,984		*n =* 8,338		*n =* 3,175	
	*M*	SD	*M*	SD	*M*	SD	*M*	SD	*M*	SD	*M*	SD
INS	4.51	(1.68)	4.86	(1.71)	4.24	(1.56)	4.64	(1.60)	3.95	(1.52)	4.36	(1.59)
AGG	5.69	(1.68)	5.68	(1.69)	5.02	(1.69)	4.68	(1.75)	4.84	(1.67)	4.30	(1.70)
EXT	5.70	(2.07)	5.84	(2.04)	6.30	(1.90)	6.44	(1.88)	5.91	(1.95)	5.66	(1.90)
MOB	4.55	(1.87)	4.70	(1.74)	4.84	(1.70)	4.61	(1.65)	4.83	(1.63)	4.59	(1.56)
VIT	5.67	(1.83)	4.92	(1.76)	6.10	(1.81)	5.24	(1.75)	6.01	(1.82)	5.22	(1.78)
ACH	5.19	(1.65)	5.19	(1.62)	5.19	(1.63)	5.48	(1.53)	5.11	(1.65)	5.45	(1.54)
RIG	4.75	(1.99)	4.87	(1.94)	5.63	(1.93)	6.08	(1.83)	5.96	(1.81)	6.46	(1.72)
EMP	4.91	(2.07)	5.91	(1.95)	5.53	(1.96)	6.53	(1.76)	5.68	(1.92)	6.49	(1.73)
DOM	4.72	(2.22)	3.95	(2.12)	4.89	(2.04)	4.49	(1.99)	4.94	(2.02)	4.67	(1.99)
SPO	5.01	(2.07)	5.13	(2.08)	4.63	(1.97)	4.65	(1.92)	4.28	(1.89)	4.19	(1.76)
OPN	5.22	(2.05)	4.85	(1.93)	4.77	(1.98)	4.05	(1.89)	4.45	(1.92)	3.69	(1.83)

Variables are explained in Table 2.

Statistics for all the following effect sizes are presented in Table 6. Contrary to our hypothesis (H1), we did not find significant differences in emotional stability between generations. Although *Instability* scores decreased, the effect sizes did not reach significance. In line with our hypothesis (H2), *Aggressiveness* showed a decrease from Generation X to Z. As for the expected increase in *Extraversion* (H3), it was only true for Generation X and Y [*F*(1,82,138) = 895.69, *p* < 0.001, *ω*^2^ = 0.012]. Due to a decrease in *Extraversion* for Generation Z, there was no substantial effect size between Generation X and Z [*F*(1,82,138) = 0.16, *p* < 0.682, *ω*^2^ = 0.000]. Despite the anticipation of higher scores in *Mobility* (H4) and *Vitality* (H5) in later generations due to the growing importance of mobility and physical fitness, the effect sizes did not reach significance. While it is suggested in the literature that conscientiousness increases across generations, we did not find a difference in *Achievement* between generations (H6). However, there was a small effect size for *Rigidity,* which increased from Generation X with the highest scores in Generation Z (H7). Today’s generations are often described as having higher self-esteem and assertiveness. We expected an increase in *Dominance* from Generation X to Z (especially for women), but this was not supported by the data (H9). Generation Z is often characterized as narcissistic with lower levels of empathy. Consequently, we expected Generation Z to be less empathetic (H8) and more demanding (H10), with higher scores in *Spoiltness* and lower scores *in Empathy*. However, for both variables, generational differences did not reach significance. Since Generation Z is described as being most familiar with self-presentation, we expected a higher level of impression management through self-presentation in a socially desirable way (reflected in scores on the TSS scale *Opennes*s). Not only did we find self-presentation to be highest in Generation Z (H11), but we also observed decreasing scores of openness from Generation X to Z.

**Table 6:** Effect sizes for differences between generations, gender and the correlations between self-presentation and TSS scales for Generation X (*N* = 20,103), Y (*N* = 50,531), and Z (*N* = 11,513).

	Generation *df* = 2,82,138			Gender *df* = 1,82,138			Self-presentation Generation		
	*F*	*ω* ^2^	Size	*F*	*ω* ^2^	Size	X	Y	Z
INS	73.69	0.001	—	1583.71	0.016	S	0.38^M^	0.36^M^	0.39^M^
AGG	747.84	0.013	S	4.35	0.000	—	0.50^M^	0.49^M^	0.48^M^
EXT	673.47	0.015	S	25.80	0.000	—	−0.12^S^	−0.10^S^	−0.11^S^
MOB	45.86	0.001	—	5.79	0.000	—	0.14^S^	0.12^S^	0.11^S^
VIT	144.83	0.004	—	2314.62	0.027	S	−0.10^S^	−0.05^−^	−0.07^−^
ACH	50.17	0.001	—	22.80	0.000	—	−0.18^S^	−0.18^S^	−0.20^S^
RIG	1008.84	0.019	S	35.63	0.000	—	−0.40^M^	−0.39^M^	−0.41^M^
EMP	360.52	0.008	—	2078.93	0.023	S	−0.09^−^	−0.11^S^	−0.10^S^
DOM	195.11	0.005	—	478.55	0.006	—	0.03^−^	0.09^−^	0.04^−^
SPO	332.87	0.008	—	36.34	0.000	—	0.19^S^	0.20^S^	0.20^S^
OPN	605.61	0.014	S	980.16	0.010	S			

Variables are explained in Table 2. Size of effect: (none)/S (small)/M (medium)/L (large). All significant with *p* < 0.001.

### 3.3. Gender differences

Independent of generation, we expected gender differences in certain variables (H12). No significant effects were found for the interaction between generation and gender, so we only present the main effects. Across all generations, women showed higher scores in emotional *Instability* and *Empathy* (Table 6). They also scored lower in *Vitality* in every generation. No differences were observed for *Extraversion* or *Mobility*. Women generally had smaller mean scores for *Dominance*, but the differences did not reach significance. Normally, women should have higher scores in conscientiousness than men, but in our sample, we did not find differences in *Achievement* or *Rigidity*.

We also observed a small gender effect in every generation, with women presenting themselves in a more socially desirable way (lower scores on *TSS-Openness*) than men.

### 3.4. Self-presentation

*Aggressiveness* was the scale most strongly influenced by self-presentation*, r*(82,145) = 0.50, *p* < 0.001, followed by *Rigidity r*(82,145) = −0.41, *p* < 0.001 and *Instability r*(82,145) = 0.38, *p* < 0.001. Applicants who described themselves in a socially desirable way (low in TSS *Openness*), were less aggressive, more rigid and less emotionally stable.

Smaller but still significant correlations showed up for *Spoiltness r*(82,145) = 0.21, *p* < 0.001, *Achievement r*(82,145) = −0.18, *p* < 0.001 *Mobility r*(82,145) = 0.12, *p* < 0.001 and *Extraversion r*(82,145) = −0.11, *p* < 0.001. The tendency for higher self-presentation (low in TSS Openness) correlated with a self-description of being less demanding (*Spoiltness*), more ambitious (*Achievemen*t), less prone to risk taking (*Mobility*) and higher in extraversion. Table 6 presents correlations for every generation.

## 4. Discussion

Our study yielded only a few results in line with generational theory, and even those lacked a clear trend. For example, *Extraversion* increased from Generation X to Y as expected (Twenge, 2001a), but the mean score for Generation Y decreased again. While conscientiousness was expected to increase across generations (Smits et al., 2011), we only found a small effect indicating that people became more rigid, but we did not find generational differences in ambitiousness. *Aggressiveness* decreased from Generation X to Z, in accordance with the results from Smits et al. (2011), but we could not confirm a generational effect of decreasing emotional instability (Twenge, 2000).

Some publications suggest that people have become more self-oriented, showing higher scores in narcissism, individualism, and lower empathy (Blok, 1998; Twenge et al., 2008, 2012a). However, in our sample, we did not find significant differences between generations. Looking at the mean scores, there seems to be a tendency for people to become more empathetic and less demanding, but this did not reach significance.

Contrary to our hypothesis, there were no higher mean scores in *Mobility* or *Vitality* and *Dominance*. While *Mobility* and *Vitality* are specific scales of the TSS questionnaire, *Dominance* is a scale also used in other questionnaires as well. Twenge reported an increase in dominance specifically for women in earlier generations (Twenge, 2001b). Although there is a lower mean score in dominance in Generation X compared to Y and Z, neither main nor interaction effects reached significant.

Certain personality traits are linked with gender. If the percentage of men or women changes in a cohort, it might influence the results of generational differences. In our study, women described themselves as less robust, less emotionally stable, and higher in empathy, which is consistent with other studies (Davis, 1980; Lippa, 2010; Vecchione et al., 2012; Lehmann et al., 2013). These differences remained stable in every generation.

One notable result of our study is the importance of self-presentation in personality measurement and indirectly in generational research. We found a high correlation between self-presentation and certain personality scales across all generations. This aligns with the findings of Khorramdel et al. (2014), who reported higher scores of “faking good” in relevant variables for pilot applicants compared to other groups. Additionally, we discovered a generational difference in self-presentation. Maybe the importance of self-presentation in today’s digital world has become more significant, and people are more familiar with it. This could be influenced by the specific context of high potential selection. Nowadays, applicants use the internet for preparation and are informed about desirable personality traits in aviation, such as stress resistance, reliability, and the importance of being outgoing. As a result, they may present themselves in a way that aligns closely with the “aviation personality” (Fitzgibbons et al., 2004). Additionally, there might be differences between men and women in different generations. Women may describe themselves in a more conformist way according to stereotypes or feel compelled to present themselves in a manner suitable for a “man’s aviation world.” In future generational research, we advise to control for self-presentation in studies.

Overall, we did not find substantial differences in personality traits between generations. The effect sizes were extremely small, and trends were sometimes unclear. For the aviation industry, there is no significant concern that applicants from Generation X or Z would differ greatly in emotional stability, conscientiousness, or personality traits related to interpersonal behavior.

However, this study has certain limitations. It is important to note that the generalization of findings to young adults in general and to other aspects of personality may be limited. The measures were taken under highly controlled conditions as part of a selection procedure with a highly homogeneous group of individuals. The study focused specifically on personality traits, and differences between generations might arise when considering attitudes, values, norms, beliefs, attention span, or psychomotor abilities. Additionally, structural invariance was not controlled due to the absence of raw scores on an item level.

Personality characteristics are a mix within all populations, and cultural influences may account for small differences between them (Hofstede and McCrae, 2004). Furthermore, trait changes over the lifespan may be influenced by culture (Chopik and Kitayama, 2018). Comparisons between generations often rely on samples from a single culture, leaving the question open as to whether differences in personality traits are limited to that specific culture (Twenge, 2001a). Even within the same culture, socioeconomic changes can also contribute to differences in personality traits, particularly in neuroticism, conscientiousness, and extraversion (Jokela and Keltikangas-Järvinen, 2011; Jonassaint et al., 2011). In our study, we only analyzed data from the German selection procedure and did not compare with other countries, limiting the generalizability of the results to one country.

For future research, it is worth questioning whether the generational approach is the most effective way to detect differences in personality traits between cohorts. While we adopted the generational approach due to its predictive nature and popularity in Human Resources (see special issue of the Journal of Business and Psychology, 2010), as well as its use by psychological organizations (American Psychological Association, 2018), the effects observed in our study were extremely small.

In accordance with other studies (Donnellan and Trzesniewski, 2009; Costanza et al., 2012; Becton et al., 2014), we conclude that there are either no differences or only negligible differences in personality traits between Generations X, Y, and Z. Some authors even doubt the explanatory power of generational theory for workplace differences altogether (Rudolph et al., 2020). It may be more fruitful for future research to focus on specific events and their impact on individual cohorts (Parry and Urwin, 2017) rather than relying solely on stereotypes (Eschleman et al., 2016; Hayes et al., 2018). Regardless of the research approach, we believe that controlling for structural invariance and social desirability should be an integral part of future studies comparing cohorts using personality questionnaires.

## 5. Conclusion

Our study identified only minor differences in personality traits between Generations X, Y, and Z. For the aviation industry, we can conclude that generational differences in personality traits do not significantly impact training and safety considerations. However, our study does not provide any insights into differences in values or abilities. We did find consistent gender differences across all generations, and a high impact of self-presentation on our measurement.

## Data availability statement

The datasets presented in this article are not readily available because of DLR data protection rules. Data is from selection procedures. Requests to access the datasets should be directed to dirk.stelling@dlr.de.

## Ethics statement

Ethical review and approval was not required for the study on human participants in accordance with the local legislation and institutional requirements. The patients/participants provided their written informed consent to participate in this study.

## Author contributions

The author confirms being the sole contributor of this work and has approved it for publication.

## Conflict of interest

The author declares that the research was conducted in the absence of any commercial or financial relationships that could be construed as a potential conflict of interest.

## Publisher’s note

All claims expressed in this article are solely those of the authors and do not necessarily represent those of their affiliated organizations, or those of the publisher, the editors and the reviewers. Any product that may be evaluated in this article, or claim that may be made by its manufacturer, is not guaranteed or endorsed by the publisher.

## Footnotes

^1^https://www.ibm.com/analytics/-spss-statistics-software
